# Emergence and increased epidemic potential of dengue variants with the NS5_V357E_ mutation after consecutive years of transmission

**DOI:** 10.1016/j.isci.2024.110899

**Published:** 2024-10-11

**Authors:** Hui-Ying Ko, Yao-Tsun Li, Han-Peng Yu, Ya-Yuan Li, Ming-Tsai Chiang, Yogy Simanjuntak, Yi-Ling Lee, Shih-Syong Dai, Pei-Jung Chung, Guann-Yi Yu, Day-Yu Chao, Yi-Ling Lin

**Affiliations:** 1 Institute of Biomedical Sciences, Academia Sinica, Taipei, Taiwan; 2 Graduate Institute of Microbiology and Public Health, National Chung-Hsing University, Taichung, Taiwan; 3 Biomedical Translation Research Center, Academia Sinica, Taipei, Taiwan; 4 Institute of Microbiology and Immunology, National Defense Medical Center, Taipei, Taiwan; 5 National Institute of Infectious Diseases and Vaccinology, National Health Research Institutes, Zhunan, Taiwan; 6 Doctoral Program in Microbial Genomics, National Chung Hsing University and Academia Sinica, Taichung City, Taiwan; 7 Department of Post-Baccalaureate Medicine, National Chung Hsing University, Taichung City, Taiwan

**Keywords:** Health sciences, Virology, Clinical microbiology

## Abstract

Arboviruses can intensify epidemics by acquiring single nucleotide variants, leading to clade replacement and severe outbreaks. We investigated dengue virus serotype 2 evolution in consecutive outbreaks from 2001 to 2003 in Taiwan, coinciding with overwintering and increased epidemic severity. The virus evolved from the early-epidemic strain (Ia) to the late-epidemic strains (Ib and II), featuring three amino acid differences. The later strains demonstrated increased replication at lower temperatures, and the NS5_V357E_ mutation significantly boosts virus replication and virulence, regardless of the other two mutations (E_T46I_ and NS5_I271T_). Crucially, the late NS5_V357E_ signature swiftly emerged after infecting mosquitos with the early Ia strain, through thoracic injection or by feeding on Ia-infected mice. Thus, we discover the molecular events involved in overwintering and increased disease severity between consecutive dengue outbreaks. This study enhances our understanding of dengue epidemiology, aiding in predicting and monitoring the emergence of dengue strains with increased epidemic potential.

## Highlights

• DENV evolved to increased epidemic potential and overwintering in Taiwan
• Mutation of NS5_V357E_ enhances dengue virus replication and virulence
• NS5_V357E_ emerged swiftly in mouse-mosquito transmission from early epidemic strain
• Study reveals molecular event connecting overwintering to severe dengue outbreaks

## Graphical abstract

**Figure:**
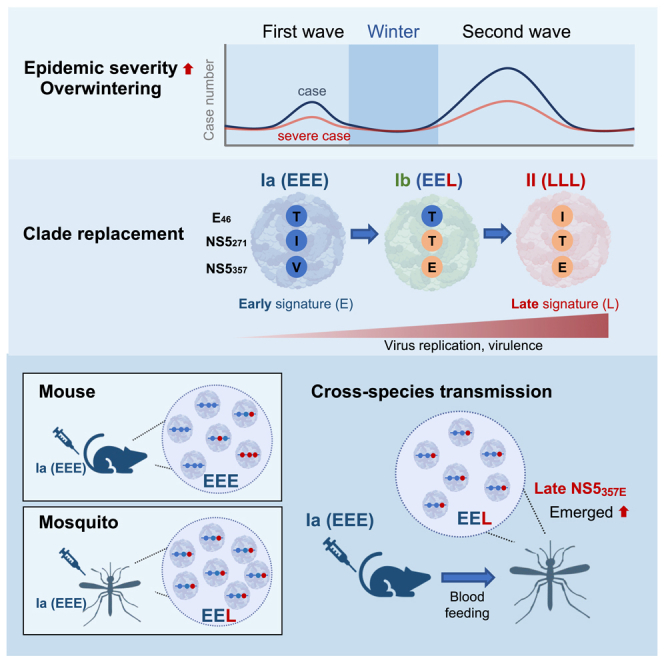

## Introduction

Dengue virus (DENV) is a widely distributed arthropod-borne virus (arbovirus) comprising four serotypes (DENV-1 to DENV-4), transmitted by *Aedes* mosquitoes, with *Aedes aegypti* as the primary vector and *Aedes albopictus* as a less efficient secondary vector. DENV is typically found in tropical and subtropical areas, but sporadic outbreaks in temperate areas have also been reported in recent decades.^1^^,^^2^^,^^3^^,^^4^^,^^5^ The DENV genome consists of three structural proteins (capsid [C], precursor membrane/membrane [prM/M], and envelope [E]) and seven non-structural proteins (NS1, NS2A, NS2B, NS3, NS4A, NS4B, and NS5). In humans, DENV infection causes a range of outcomes, from asymptomatic infection to severe dengue.^6^ Severe dengue is a significant cause of hospitalization and mortality in endemic countries, and poses substantial challenges to public health and clinical management.^7^

It has been reported that arboviruses, such as West Nile virus (WNV), DENV, Japanese encephalitis virus (JEV), and Chikungunya virus (CHIKV), can increase their epidemic potential by acquiring even a small number of single nucleotide variants (SNVs), which can lead to clade replacement and the emergence of severe outbreaks.^8^^,^^9^^,^^10^^,^^11^^,^^12^ Previous studies indicate that clade replacement events result from a complex interplay of factors. Genetic mutations enhance the replicative fitness and infectivity of dengue virus strains, facilitating their dominance.^13^^,^^14^^,^^15^ Additionally, dengue subgenomic RNA modulation^16^ and vector-mediated natural selection favor strains with superior mosquito infectivity, perpetuating their prevalence.^17^ These viruses rapidly adapt to changing environments through dynamic processes like adaptive evolution. This allows them to thrive in new conditions, evade host immune defenses, and potentially emerge as new strains with increased epidemic potential. “Epidemic potential” refers to a pathogen’s capacity to initiate an outbreak, and is typically associated with a larger number of infections and/or more severe cases. This is influenced predominantly by how effectively the virus spreads between human hosts. Arboviruses exhibit genetically diverse populations, with their diversity driven by the high mutation rates of their RNA-dependent RNA polymerase (RdRP, NS5), and these populations of numerous variant genomes are referred to as mutant swarms or quasispecies.^18^ When quasispecies are transmitted between different host species involving arthropod vectors, population bottleneck occurs leading to dramatic reduction in population diversity.^19^^,^^20^ This bottleneck effect results in sub-populations of arboviruses with greater epidemic potential that may become dominant, leading to clade replacement^21^^,^^22^ and/or alterations in pathogenesis in humans.^23^ How these subpopulations are selected and the specific factors that contribute to increased epidemic severity are not yet fully understood.

Unlike many Southeast Asian countries where multiple dengue serotypes circulate simultaneously, Taiwan, situated in a subtropical area, primarily encounters dengue viruses imported from Southeast Asia.^24^ These imported cases lead to annual local outbreaks, which typically diminish in winter as mosquito populations decline.^25^ During the consecutive DENV-2 outbreaks in Taiwan between 2001 and 2003, caused by the cosmopolitan genotype, clade replacement occurred concurrently with an overwintering event. The virus transitioned from the initial Ia clade to the subsequent Ib and II clades.^10^ This shift in clades was observed after transmission within the human population and was followed by a second, larger outbreak after the winter period. This second wave had a higher number of cases (5,336 vs. 227 cases in the earlier wave) and was associated with more severe cases.^26^ Through deep sequencing, we detected minor variants with late-epidemic signatures (genetic markers of the clade II strain) in the virus quasispecies from individuals infected with clade Ia.^10^ This sheds light on the ancestral role of the early-epidemic Ia strain. Nonetheless, the causes of increased epidemic severity, the benefits of selective subpopulations, and the biological characteristics related to surviving over the winter season still require further investigation.

To evaluate the replication and virulence of these DENV-2 epidemic strains (Ia, Ib, and II), we used infectious clone technology to delve deeper into the mechanisms governing virus transmission and overwintering. We identified critical amino acid changes that impact viral loads, transmission, and DENV persistence in low-temperature environments. We also conducted cross-species experiments to track the frequency changes of these variants during transmission, providing additional insights into the virus evolution. Utilizing animal experiments and deep sequencing tools, we were able to recapitulate evolutionary outcomes in the pathogen genome within a short time span, enabling early assessments before the next outbreak. Our study sheds light on the complex dynamics of DENV epidemics and provides valuable insights into viral replication, virulence, and evolution within different hosts.

## Results

### Enhanced replication of late-epidemic DENV strains (clade II and Ib) compared to the earlier parental strain (clade Ia)

**Figure 1:**
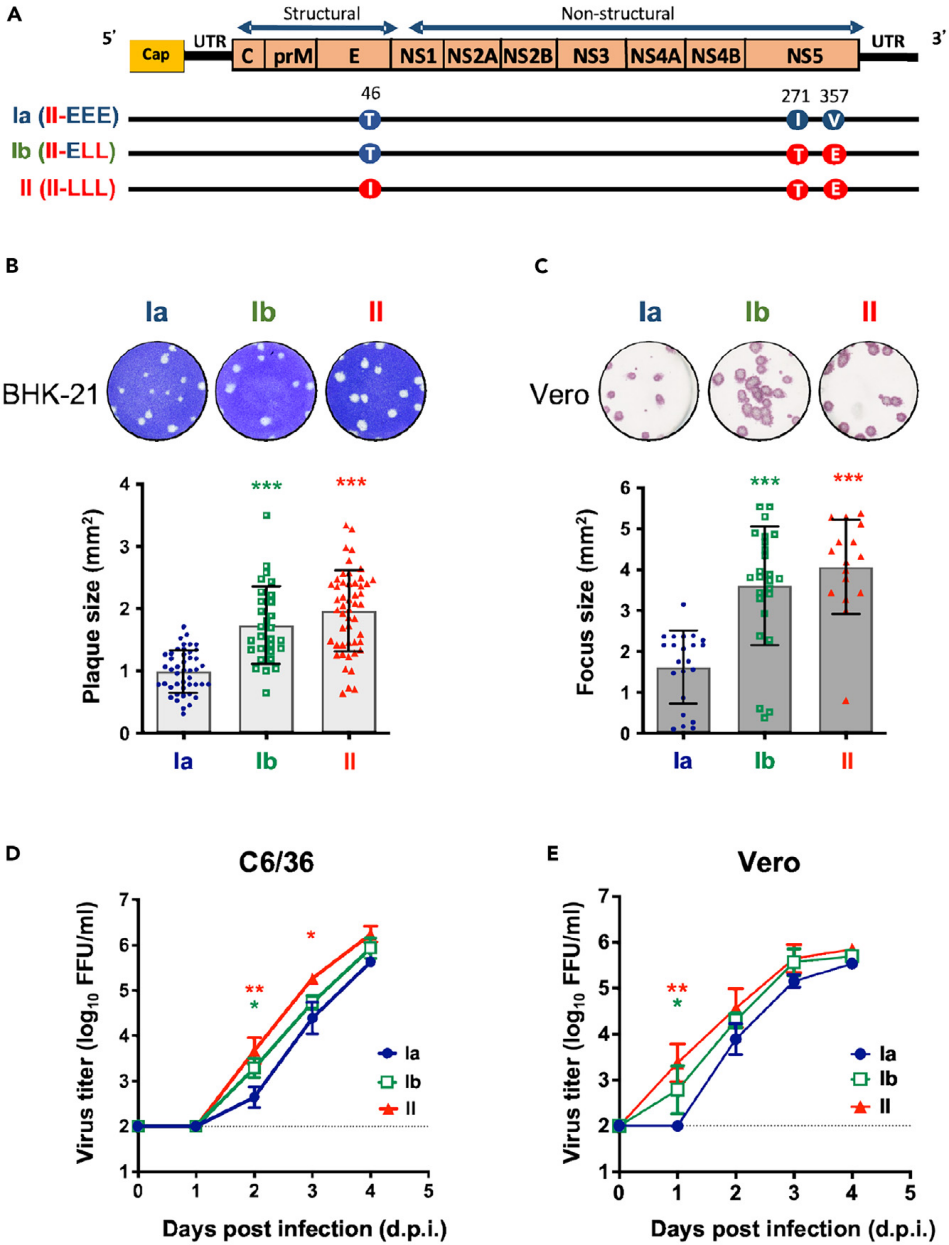
Late-epidemic DENV-2 strains (clade II and Ib) exhibit higher replication than the early-epidemic strain (clade Ia) in cells (A) Illustration of the amino acid variations among the three infectious clone-derived DENV-2 representing clade Ia, Ib, and II. Amino acid signatures associated with the early epidemic Ia are labeled as “E”, while those associated with the late epidemic II are labeled as “L”. Specifically, at the first variation site (E_46_), threonine (T) is denoted as “E”, and isoleucine (I) is denoted as “L”. At the second position (NS5_271_), isoleucine (I) is labeled as “E”, and threonine (T) is labeled as “L”. Finally, at the third position (NS5_357_), valine (V) is designated as “E,” and glutamine (E) is designated as “L.” For clarity, Ia was denoted as II-EEE, Ib as II-ELL, and II as II-LLL. (B and C) To study the significance of these amino acid changes, Ia, Ib, and II viruses were reconstructed using the clade II virus as a backbone. We performed quantitative analysis of the plaque size (B) and focus size (C) of these viruses using ImageJ software. At least 15 plaques or foci were selected for size analysis from an appropriate concentration in the plaque assay or focus-forming assay. Plaque/foci sizes are represented as mean ± standard deviation (SD). The upper panels illustrate the representative morphology of virus plaques and foci. (D and E) The growth kinetics of Ia, Ib, and II viruses in mosquito (C6/36) cells (D) and in mammalian (Vero) cells (E) with a multiplicity of infection (MOI) of 0.1. Virus titer data are presented as the mean and SD. Statistical significance is denoted by asterisks, where red indicates significant differences between clade II and Ia, and green indicates significant differences between clade Ib and Ia, as determined by one-way ANOVA tests. Significance levels are denoted as ∗*p* < 0.05, ∗∗*p* < 0.01, and ∗∗∗*p* < 0.001.

During the consecutive dengue epidemics that occurred in Taiwan from 2001 to 2003, the clade Ia strain served a pivotal role as the parental strain, inciting the initial wave of the epidemic in 2001.^10^ The late-epidemic strains, clade Ib and II, were predominantly associated with the overwinter period and the subsequent severe epidemic wave, which began in 2002 with more clinically severe cases. Sequence analysis of DENV-2 isolates across these three clades revealed a total of 12 nucleotide changes, resulting in three non-synonymous mutations - one at position 46 of the E protein (T vs. I), and two at positions 271 (I vs. T) and 357 (V vs. E) of the NS5 protein (Figure 1A).

Due to the significant changes in these amino acids, we investigated the replication dynamics of these three epidemic DENV strains in relation to these amino acid mutations. We utilized infectious clones to reconstruct these DENVs, using the clade II strain as a backbone. The late-epidemic signatures (E_46I_, NS5_271T_, and NS5_357E_) are represented as “L” and the early-epidemic signatures (E_46T_, NS5_271I_, and NS5_357V_) are represented as “E”. Thus, the infectious clone-derived viruses with the Ia amino acid signatures are denoted as Ia (II-EEE), Ib as Ib (II-ELL), and II as II-LLL (Figure 1A). To evaluate the replication of these three DENV strains, we analyzed the foci and plaque morphology by infecting Vero and BHK-21 cells, respectively. The reconstructed Ib and II viruses formed larger foci and plaques than the Ia virus (Figures 1B and 1C). Moreover, when compared to the Ia virus, the Ib and II viruses replicated to significantly higher titers during the early stages of infection in both C6/36 and Vero cells (Figures 1D and 1E).

### The NS5_357E_ variant enhances virus replication of the late-epidemic DENV strains in cells

**Figure 2:**
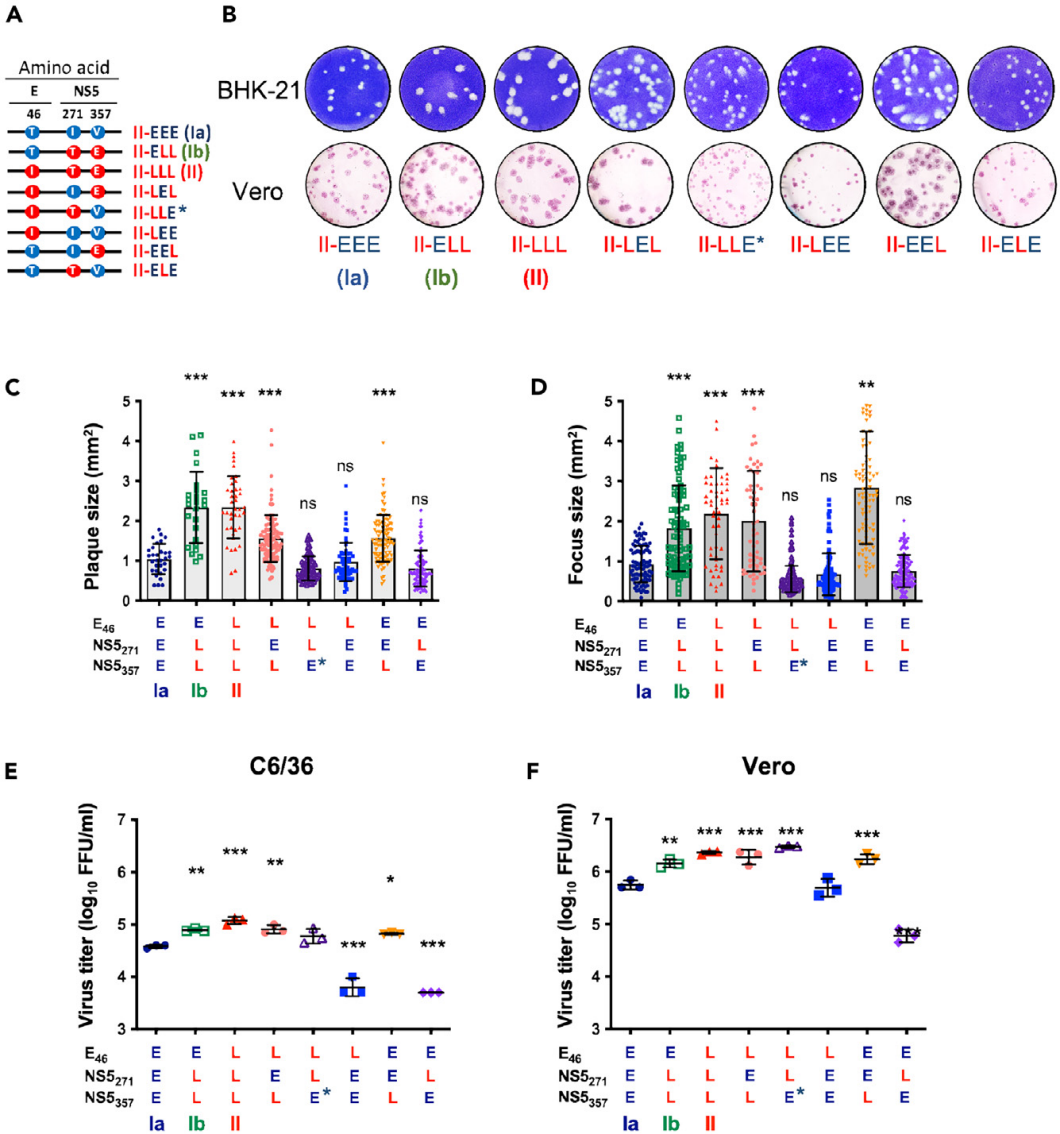
The late epidemic signature NS5_357E_ exhibits enhanced virus replication in both mosquito and mammalian cells (A) Illustration of the amino acid variations among the eight infectious clone-derived DENV-2 viruses, covering all possible combinations of the three amino acid signatures (E_46_, NS5_271_, and NS5_357_). We marked the II-LLE strain as II-LLE∗ to reflect its mixed population. (B) Plaque morphology (upper panel) and focus morphology (lower panel) of viruses carrying various combinations of mutations were assessed in BHK-21 and Vero cells, respectively. (C and D) For quantitative analysis, we selected at least 15 plaques or foci, and measured their size using ImageJ software. The results are presented in the bar chart showing plaque size (C) and focus size (D), with plaque/focus sizes represented as mean ± SD. (E and F) We evaluated the growth of DENV-2 with different combinations of mutations at MOI 0.1 in C6/36 (E) and Vero (F) cells. Virus titers at 2 days post-infection (d.p.i.) are presented as the mean and SD in the jitter plots. Statistical analysis was performed using one-way ANOVA to determine the differences between Ia and the mutant viruses, with significance levels denoted as ∗*p* < 0.05, ∗∗*p* < 0.01, and ∗∗∗*p* < 0.001.

To identify the amino acid substitution contributing to the enhanced virus replication in late-epidemic DENV strains, we generated five additional infectious clones covering all possible combinations of the three amino acid signatures (Figure 2A). All of these DENV strains can be generated by infectious clones and exhibited competent replication in both mammalian and mosquito cells. To evaluate their replication efficiency, we analyzed the plaque and foci morphology in BHK-21 and Vero cells, respectively. DENV-2 viruses with the late-epidemic signature at the third position (NS5_357E_) (II-ELL, II-LLL, II-LEL, and II-EEL) exhibited significantly larger plaque and focus sizes than the parental Ia (II-EEE) virus (Figures 2B–2D). Furthermore, all viruses with NS5_357E_ replicated to higher titers when compared to the Ia virus in both C6/36 and Vero cells, regardless of the signature at the first and second positions (Figures 2E and 2F).

Through pyro-sequencing, we noticed a mixed population in the viral stock of the II-LLE variant at the third position of NS5_357_, with 57% showing valine (early signature) and 43% showing glutamic acid (late signature). The NS5_V357E_ mutation emerged in the viral population following plasmid transfection into 293T/17 cells (P0). We marked the attempted II-LLE strain as II-LLE∗ (Figure 2) to reflect its mixed population. Interestingly, II-LLE∗ displayed a phenotype more similar to that of the II (II-LLL) strain, probably due to the enhancing effect of NS5_357E_ on viral replication (Figures 2E and 2F).^27^ Taken together, these findings suggest that the V357E mutation on NS5 in infectious clone-derived strains enhances virus replication.

### Clade replacement during DENV-2 outbreaks driven by a virus replication advantage in the clade II virus

**Figure 3:**
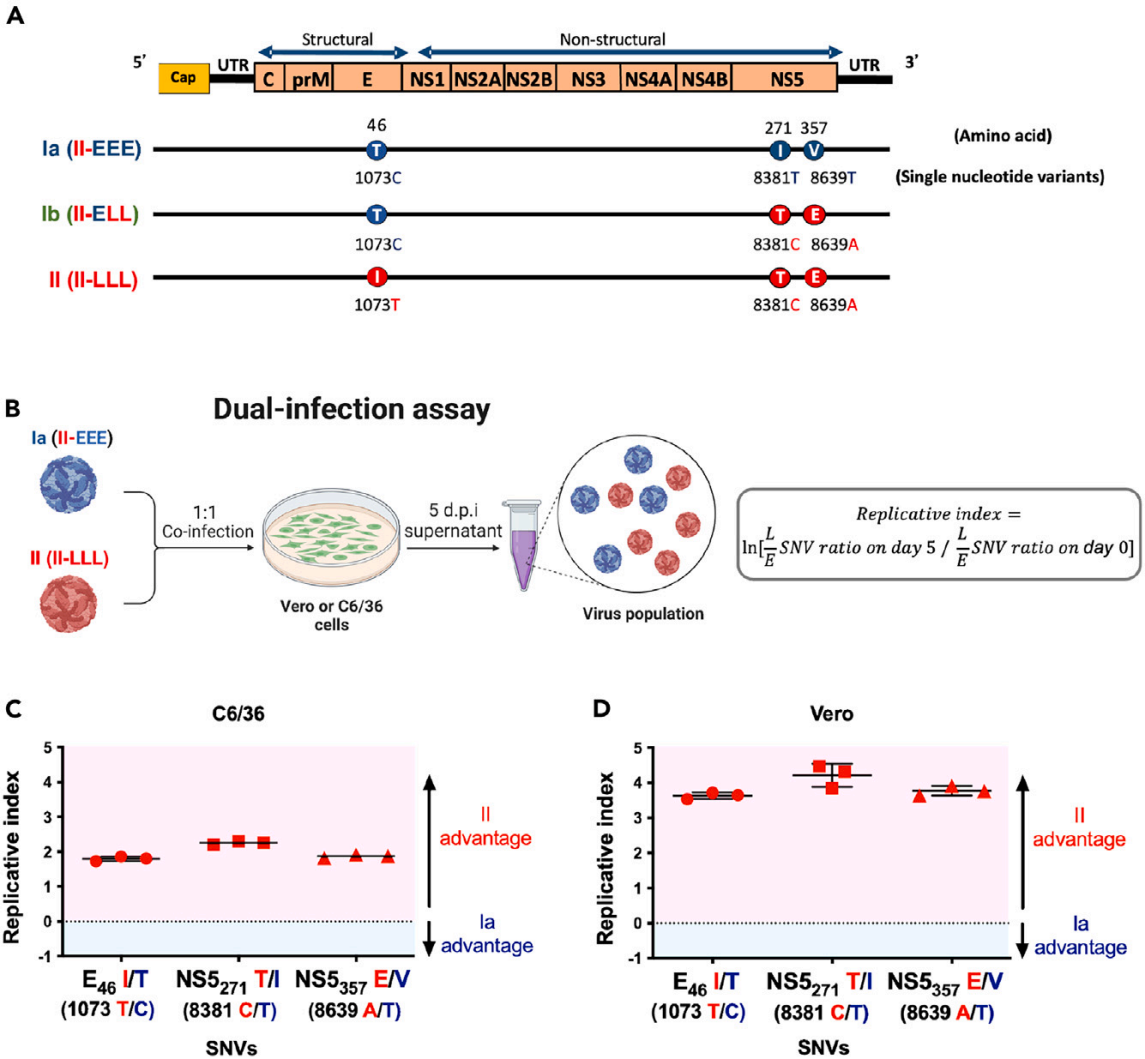
The late-epidemic clade II virus exhibited a higher virus replication advantage than Ia in both mosquito and mammalian cells (A) Illustration of the three amino acid signatures of the DENV-2 epidemic strains at E_46_, NS5_271_, and NS5_357_ as well as their correlated changes in single nucleotide variants (SNVs), specifically SNV-1073, SNV-8381, and SNV-8639. (B) To evaluate the relative fitness of clade Ia and II, dual-infection assays were conducted in both C6/36 and Vero cells. We used a 1:1 mixture of Ia (II-EEE) and II (II-LLL) viruses to infect cells with an MOI of 0.5. We collected supernatants at 5 d.p.i., and the proportion of SNVs was determined through pyrosequencing. We analyzed and presented the data as a replicative index, as depicted in the equation on the right, which quantifies the relative change of each clade from its initial value. (C and D) The replicative index of the clade Ia virus in competition with the clade II virus in C6/36 (C) and Vero (D) cells, presented as the mean ± SD. A replicative index of 0 indicates equal fitness of the indicated SNVs as represented by dotted lines. A replicative index >0 indicates a clade II advantage, while an index <0 indicates a clade Ia advantage.

During the DENV-2 epidemics in Taiwan from 2001 to 2003, a clade replacement was observed.^10^ To investigate the underlying viral mechanisms, we conducted dual-infection assays on Vero and C6/36 cells to evaluate the relative replication capacity of the early (Ia, II-EEE) and the late (II, II-LLL) epidemic strains (Figure 3B). Using pyrosequencing to monitor changes in the proportion of SNVs corresponding to the late-epidemic signatures (as illustrated in Figure 3A), we calculated the replicative index to evaluate the relative replication efficiency of clades Ia and II. The replicative index quantifies the relative change in the ratio of late-epidemic SNVs (L SNVs) to early-epidemic SNVs (E SNVs), compared to its initial value (Figure 3B). When the dual-infection was performed with Ia and II viruses at a ratio of 1:1, the replicative index at all three positions were found to be greater than 0 in both C6/36 and Vero cells (Figures 3C and 3D), indicating that the clade II virus was able to outcompete the earlier clade Ia. These observations fit in the context of clade replacement during the outbreaks in which the late-epidemic strains replaced the early-epidemic strain.

### The NS5_357E_ variant confers low temperature adaptation to late-epidemic DENV-2 strains in mosquito cells

**Figure 4:**
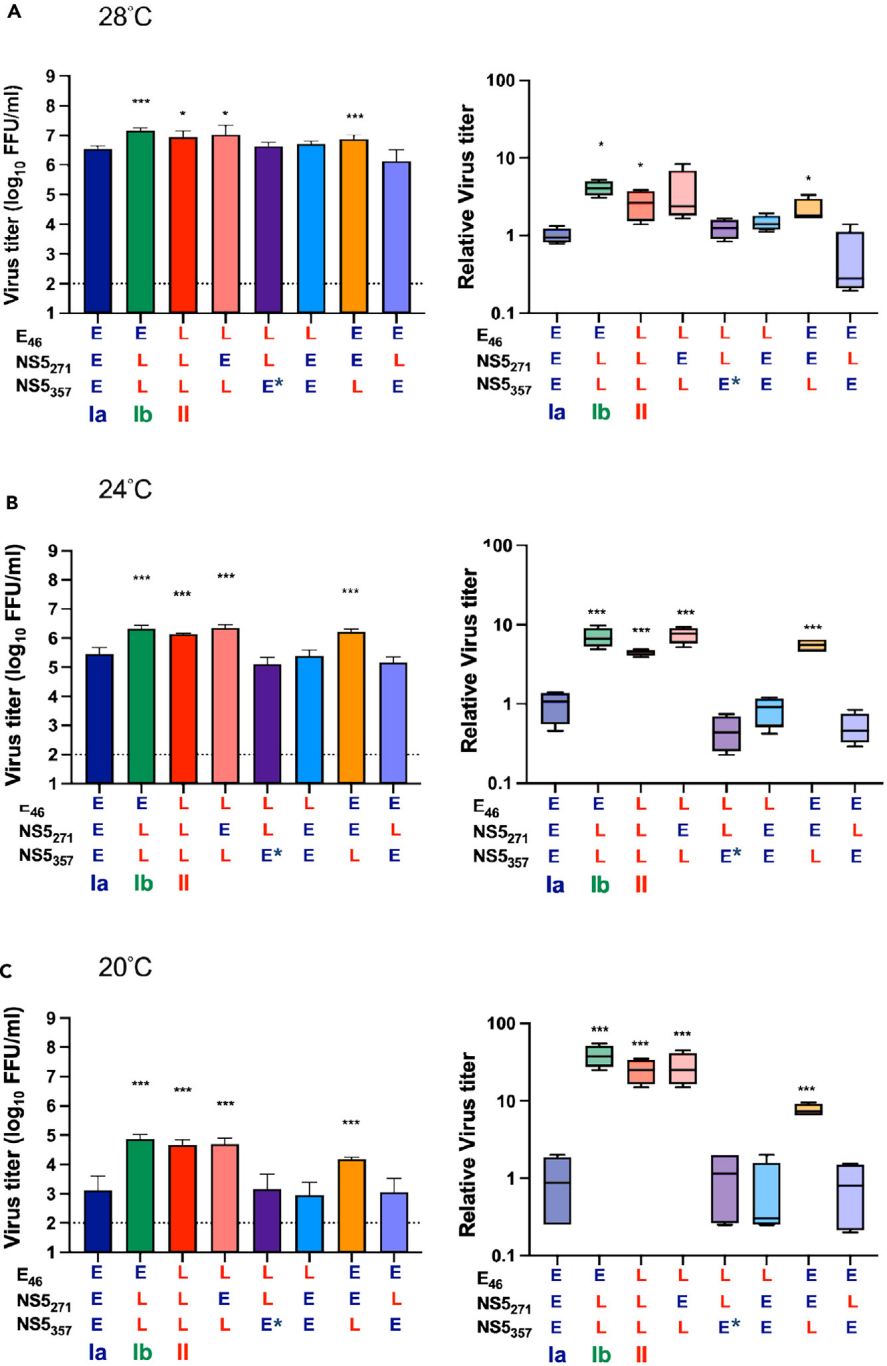
The NS5_357E_-carrying viruses exhibit increased virus replication advantages at low temperatures We assessed the virus replication capacity of infectious clone-derived DENV-2 viruses carrying various combinations of early/late epidemic signatures in C6/36 cells at different temperatures, including 28°C (A), 24°C (B), and 20°C (C). The cells were infected with DENV-2 viruses at a MOI of 0.1, and culture supernatants were collected at 4 d.p.i. All experiments were performed with four independent replicates. Virus titers are presented as means ± SDs in the left panels (bar charts). Statistical analysis comparing each variant with Ia was performed using one-way ANOVA. Relative viral loads, compared with the parental Ia virus, are illustrated as means and ranges in the right panels (boxes). Differences between each variant and Ia were assessed using Student’s t test. Significance levels are indicated as ∗*p* < 0.05, ∗∗*p* < 0.01, and ∗∗∗*p* < 0.001.

To investigate the low-temperature adaptation of DENV-2 during the winter of 2001–2002,^10^ we examined whether the late epidemic strains replicate more efficiently in mosquito cells at low temperatures than their parental strain (Ia, II-EEE). We infected C6/36 cells with infectious clone derived viruses and cultured them at 20°C, 24°C, and 28°C. The results revealed that both Ib (II-ELL) and II (II-LLL) viruses replicated to higher titers than the Ia virus in all three temperature conditions at 4 d.p.i. (Figure 4, left panels). Interestingly, the growth advantage of Ib and II was much more pronounced at lower temperatures. At 20°C, viral production of Ib and II was 38.75 and 25-fold higher than that of Ia, while at 24°C/28°C the difference was reduced to 4.08/6.95 and 2.64/4.45-fold, respectively, when compared to the Ia virus (Figure 4, right panels).

In addition, II-LEL and II-EEL viruses carrying the NS5_357E_ late epidemic signature, regardless of the changes at E_46_ and NS5_271_, replicated to higher titers than the Ia virus at all three temperatures (left panel of Figure 4). The advantage of NS5_357E_-carrying viruses over Ia was more striking at 20°C, ranging from 7.63 to 38.75 times greater, compared to 24°C (4.45–7.48 times) and 28°C (2.15–4.01 times). Thus, the NS5_V357E_ mutation in the late epidemic strains plays a key role in sustaining virus replication during the winter time.

### The NS5_357E_ variant showed replication advantages of late-epidemic strains in AGB6 mice

**Figure 5:**
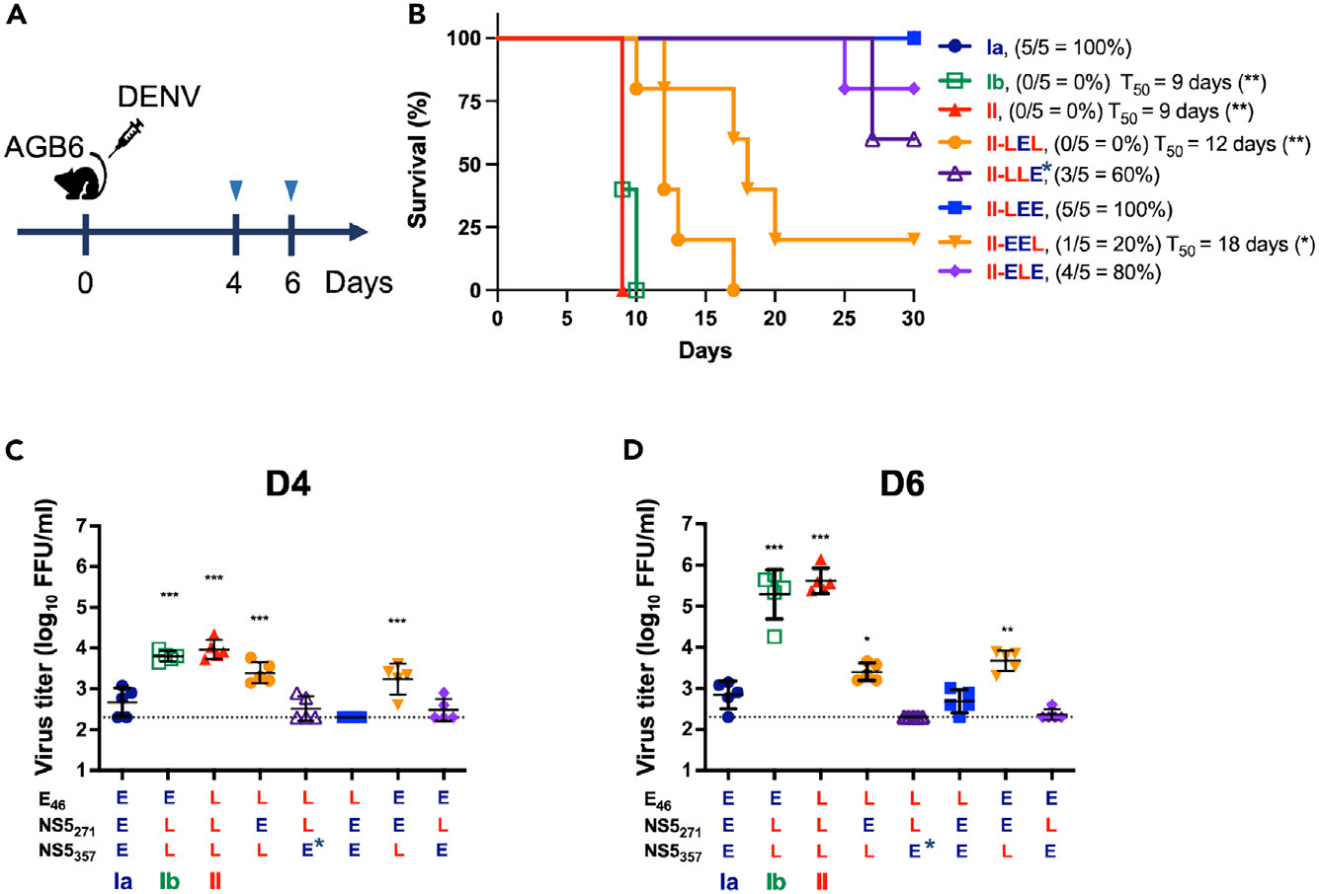
DENV-2 strains carrying the late epidemic NS5_357E_ variant exhibited higher virulence in AGB6 mice (A) AGB6 mice were infected with 500 PFU of the infectious clone-derived viruses (*n* = 5 for each group) by footpad injection. To assess the virulence of infectious clone-derived viruses, their impact on AGB6 mice was evaluated through survival (B) and viral load measurements in serum collected at 4 d.p.i. (C) and 6 d.p.i. (D). Virus titers are represented as mean ± SD. Survival analysis was performed with Kaplan-Meier curves to compare the survival difference between each group and the Ia-inoculated group according to survival rate and medium survival time (T_50_). Significance was indicated as ∗*p* < 0.05, ∗∗*p* < 0.01. Statistical analysis for differences in virus titers in mouse serum between Ia and the mutant viruses was conducted using one-way ANOVA, with significance indicated as ∗*p* < 0.05, ∗∗*p* < 0.01, ∗∗∗*p* < 0.001.

To further understand the replication and pathogenesis of these DENV-2 strains in mammalian hosts, we inoculated 500 PFU of the infectious clone-derived viruses into AGB6 mice via footpad injection and monitored their survival rates and viral load in mouse sera (Figure 5A). All mice inoculated with the late-epidemic Ib and II strains died by 10 d.p.i., whereas all of the Ia-infected mice survived throughout the experiment (Figure 5B). We detected higher virus titers in the sera of mice infected with the late-epidemic strains compared to those infected with the Ia strain at 4 and 6 d.p.i. (Figures 5C and 5D). These findings demonstrate that the late-epidemic strains replicated to higher levels and were more virulent when compared to the Ia strain.

**Figure 6:**
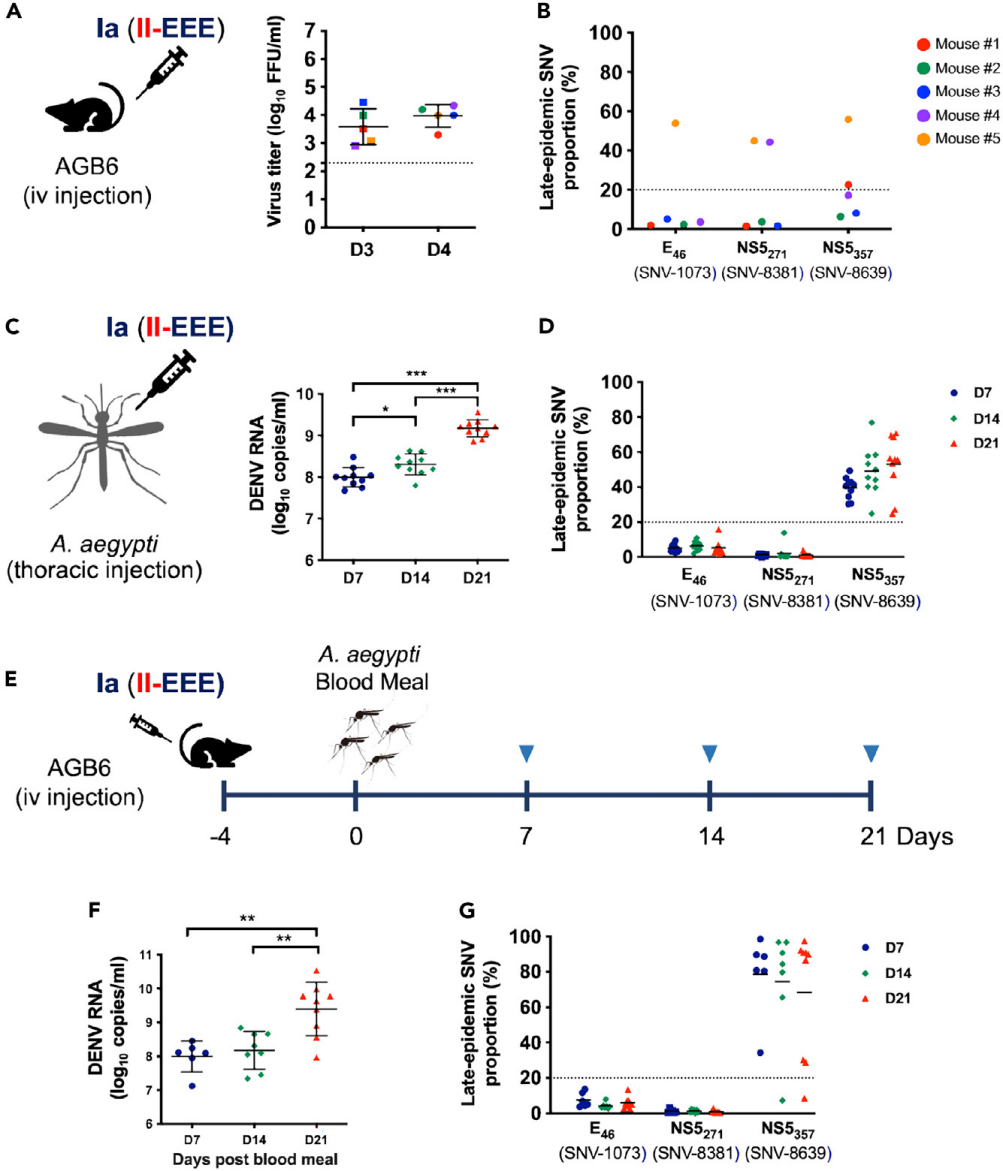
Emergence and transmission of the SNV-8639A (NS5_357E_) variant in mosquitoes, mice, and across species To monitor the emergence of the late-epidemic signatures, mice (A and B) and mosquitoes (C and D) were inoculated with the parental Ia (II-EEE) virus. We infected AGB6 mice with 500 PFU of the Ia virus via tail vein injection (*n* = 5), and we determined virus titers in mouse sera collected at 3 and 4 d.p.i. using focus-forming assays (A). The proportions of the late SNVs in each mouse at 4 d.p.i. were analyzed by pyrosequencing (B). We inoculated *Aedes Aegypti* mosquitoes with 500 PFU of the Ia virus via thoracic injection (*n* = 10 at each time point). Viral RNA copies (C) and proportions of the late SNVs (D) were determined in mosquitoes collected at 7, 14, and 21 d.p.i. (E) To monitor the emergence and transmission of late-epidemic signatures during the mice-mosquito transmission chain, we infected AGB6 mice with 500 PFU of the Ia virus and used them to feed naive mosquitoes (*n* = 12 at each time point) at 4 d.p.i. Viral RNA copies (F) and proportions of the late SNVs (G) in infected mosquitoes were determined at 7, 14, and 21 d.p.i. Virus titers and Viral RNA titers are represented as mean ± SD, while proportions of the late SNVs are expressed as the mean. Statistical significance was determined using one-way ANOVA, with significance levels indicated as ∗*p* < 0.05, ∗∗*p* < 0.01, ∗∗∗*p* < 0.001.

To identify the amino acid corresponding to high virulence, we assessed five more infectious clone-derived viruses via footpad injection into AGB6 mice. Correspondingly, virus strains carrying the NS5_357E_ late epidemic signature were more virulent (Figure 5B) and resulted in higher viral loads in challenged mice at 4 d.p.i. (Figure 5C) and 6 d.p.i. (Figure 5D) as compared with other variants. Thus, NS5_357E_ also plays a critical role in the virulence and replication of late-epidemic DENV strains within mammalian hosts.

### Emergence and propagation of the NS5_357E_ variant during a cross-species transmission model

Viruses with high mutation rates can generate a diverse quasispecies that constantly adapts to the changing environment, potentially resulting in the emergence of novel epidemic or pandemic strains. Furthermore, arboviruses, such as DENV, have the capability to undergo cross-species transmission, which can greatly affect the composition of the quasispecies within a host.^28^^,^^29^ To investigate the process of selecting new variants, we specifically examined the emergence of the three late epidemic amino acid signatures in mice (Figure 6A) and mosquitos (Figure 6C) after inoculation with the early Ia (II-EEE) strain. Three of the five Ia-infected mice showed the emergence (defined as the SNV detected in over 20% of the virus population) of the late-epidemic signatures (Figure 6B). Mouse #5 exhibited the emergence of all three late-epidemic signatures, while mouse #4 and mouse #1 only showed the emergence of the second (SNV-8381C, NS5_271T_) and third (SNV-8639A, NS5_357E_) late-epidemic SNVs, respectively. Among these mice, only mouse #5 showed minor variants at the first (SNV-1073T, E_46I_) and third late-epidemic SNVs, which emerged and became dominant (>50%). In contrast, all of the mosquitoes inoculated with Ia (II-EEE) by thoracic injection showed the emergence of the third (SNV-8639A, NS5_357E_), but not the first two (SNV-1073T, E_46I_ and SNV-8381C, NS5_271T_) late-epidemic signatures (Figure 6D). In mosquitoes injected thoracically, only the third position late variant became dominant. By day 14, it reached 50% dominance, and by day 21, it increased to 70%.

To further mimic the cross-species transmission of DENV, we fed Ia (II-EEE)-inoculated mice at 4 d.p.i. to mosquitos (Figure 6E). Similar to the intrathoracically inoculated mosquitos, the third late-epidemic SNV (SNV-8639A, NS5_357E_), but not the other two SNVs, emerged in mosquitos as early as 7 days post blood feeding (Figure 6G). Notably, the proportion of the third late SNV in each individual mosquito was even more pronounced, with approximately 80% of mosquitoes showing dominance of the late variant in the cross-species transmission model (Figure 6G). This is in contrast to the intrathoracically injected mosquitoes (Figure 6D), where only about 50% showed dominance of the late variant. Our findings reveal that mosquitoes are a major host for the emergence of the dengue SNV-8639A (NS5_357E_) variant. This variant confers a growth advantage, and passing through infected mammals may enhance the emergence of late-epidemic variants in mosquitos.

## Discussion

During the DENV-2 epidemics that took place in Taiwan between 2001 and 2003, clade replacement occurred, with the parental Ia strain evolving into the late-epidemic strains (Ib and II), which were associated with both increased epidemic severity and virus overwintering.^30^ Our dual-infection assays indicate that the virus with the late-epidemic signature outcompeted the early-epidemic strain (Figure 3), suggesting a possible underlying mechanism for clade replacement observed in the epidemiological studies.^10^^,^^26^ Furthermore, the 2002 outbreak surpassed the 2001 outbreak in scale, with a notable rise in severe cases, from 6.3% to 7.7% DHF cases.^26^ Our animal study showed that AGB6 mice infected with the late-epidemic DENV-2 had higher blood titers and mortality rates compared to those infected with the early-epidemic strain (Figure 5), in consistent with the reports that blood titers reflect viral replication kinetics and disease severity in dengue patients.^31^^,^^32^^,^^33^

In this study, we replicated the emergence of the NS5_357E_ variant, which is a late-epidemic signature with a growth advantage in multiple settings, including mosquitoes, AGB6 mice, and the mouse-mosquito transmission model. The NS5_V357E_ mutation significantly boosts DENV-2 replication and virulence. The replication advantages of NS5_357E_ increase the likelihood of new variants being selected during transmission, similar to the mechanism observed in CHIKV, a vector-borne alphavirus, from which also emerged intermediate evolutionary strains for causing epidemics.^34^ Furthermore, mosquitoes are the key hosts involved in the emergence of the NS5_357E_ variant, highlighting the crucial role of mosquitoes as key mediators in the ecological and epidemiological aspects of dengue virus (Figure 6). This study highlights the significance of specific mutations, notably NS5_V357E_, in the adaptation, evolution, and severity of dengue virus epidemics. Additionally, this study replicates the emergence of a crucial residue relevant to epidemic flavivirus strains in the setting of experimental mouse-mosquito transmission. Our findings suggest that early risk assessment of circulating pathogens can be used to inform current control measures and future preventive strategies.

Although in our experimental conditions the parental Ia virus quickly acquired the late SNV (NS5_357E_), the late epidemic viruses did not emerge swiftly during epidemic in Taiwan. During the 2001 outbreak, Ia circulated and caused small outbreaks for about four months without mutating to Ib.^10^ This discrepancy may be due to laboratory conditions not fully capturing environmental factors such as temperature fluctuations and humidity.^35^^,^^36^^,^^37^^,^^38^^,^^39^ Besides, mosquito abundance, feeding habits, lifespan, and transmission settings (sylvatic or urban) could also influence SNV acquisition rates.^40^^,^^41^^,^^42^^,^^43^

Understanding how arboviruses, such as dengue, adapt to different temperatures is crucial to comprehending virus overwintering, and has significant public health implications. Global warming has the potential to increase transmission rates, making the study of temperature adaptation in arboviruses paramount.^44^ Studies have shown that temperature-sensitive mutations in the non-structural proteins of the dengue virus genome affect the virus’s sensitivity to temperature changes.^45^^,^^46^ These mutants exhibit different levels of complementation and RNA production at various temperatures. The dengue epidemic studied in this report involved an overwintering event, in which the virus persisted during the winter of 2001–2002 and continued to transmit among the human population through mosquitoes.^30^ This occurred when the average daily temperature was 20°C.^47^ To elucidate the mechanisms underlying this overwintering event, we tested the replication of dengue virus strains at 20°C, 24°C, and 28°C (Figure 4). The late-epidemic strains exhibited better replication capabilities than the early-epidemic strain, particularly at lower temperatures, underscoring their endurance even in cold conditions. This endurance likely benefited their population maintenance during the winter, when vector density and biting behavior decreased. After the winter, the virus population expanded, and viral variants with higher replication or fitness were selected, leading to the next wave of the outbreak.

During 2001–2003 in Taiwan, we observed two consecutive waves of DENV-2 epidemic and noted a temporal pattern of three late-epidemic amino acid signatures at an epidemiological scale. Specifically, the two late-epidemic signatures in the NS5 protein (NS5_271T_ and NS5_357E_) occurred in the first wave virus isolates of the Ib clade, while the E_46I_ variant emerged later during the second wave.^10^ An increase in the NS5_357E_ variant during infection of mosquitoes with the early epidemic strain (Ia, II-EEE) (Figure 6) suggests that NS5_357E_-carrying viruses have an advantage through frequent transmission between hosts, making it a “generalist” mutation. The NS5 protein consists of two domains, the methyltransferase (MTase) and RdRp domains.^48^ NS5_271_ and NS5_357_ are situated in two distinct inter-domain regions (residues 264–273 and 349–358) of the NS5 protein, respectively. These regions determine the flexibility of the NS5 protein, which can potentially impact its enzymatic activity.^27^ Thus, alterations in the NS5_357E_ variant may enhance the activity of the NS5 protein, thus promoting DENV replication in mosquitoes and mice. NS5_271T_ may not contribute to the growth advantage and overwintering, since the variants II-LLL and II-LEL, which differ only at the second position NS5_271_, replicated to similar levels in C6/36 and Vero cells, even at lower temperatures (Figures 2E, 2F, and 4). However, the late signature NS5_271T_ emerged in two of the Ia-infected mice (Figure 6B), indicating the probable beneficial effect of NS5_271T_ in mammalian hosts. Overall, these observed mutations and the growth advantage in the NS5 protein may have contributed to the clade replacement phenomenon observed during the DENV-2 epidemics.

Another late-epidemic signature E_46I_ is located in the envelope protein, which is a structural component of the virus. Epidemiological observations have revealed that viruses carrying the E_46I_ variant are selected during extensive circulation in humans.^10^ However, the rise of the E_46I_ variant was only observed in one of the infected AGB6 mice (Figure 6B), and not at all in mosquitos (Figures 6D and 6G). The substitution of threonine with isoleucine results in the replacement of a polar residue with a non-polar one, which has been suggested to stabilize the protein structure.^49^ Additionally, E_46I_ has been associated with interference in the binding of neutralizing antibodies^50^ and T cell recognition.^51^ These findings correspond to a scenario in which SNVs among viral populations derived from humans mainly occur in structural proteins, most likely through immune selection, while mosquito-derived variations occur in non-structural proteins and untranslated regions, most likely through growth advantages.^28^^,^^29^

In this study, we characterized the replication and virulence of three dengue epidemic strains and gained insights into the mechanisms driving DENV transmission, overwintering, and adaptive evolution. The assessment of epidemic potential involved investigating viral transmissibility^52^ and pathogenesis^53^^,^^54^ using experimental models. Regular evaluations of viral epidemic potential and early risk assessment of circulating pathogens could, therefore, provide valuable information for current control measures and future preventive strategies.

### Limitations of the study

Larger plaque or focus sizes usually indicate higher viral virulence and replication rates.^55^^,^^56^^,^^57^^,^^58^ Our study used plaque and focus sizes in Vero and BHK-21 cells to assess viral replication, but not in C6/36 cells due to their irregular foci shapes.^59^ While these sizes generally correlate with higher virulence, they are not definitive indicators.^58^ Thus, we also examined growth activity and survival in AGB6 mice to comprehensively understand viral pathogenicity. Another limitation is that we did not examine the effect of synonymous mutations among the three clade epidemic strains. Although these synonymous mutations do not change protein sequences, they can affect viral RNA structure, translation efficiency, RNA interactions, processing, and immune evasion, potentially influencing viral evolution and behavior.^60^^,^^61^ The third limitation is the reliance on Vero cells, chosen for their high susceptibility to DENV and widespread use in research. However, using primary human cells like monocytes and dendritic cells would afford a more physiological relevant context for DENV infection in humans.

## Resource availability

### Lead contact

Further information and requests for resources and reagents should be directed to and will be fulfilled by lead contact, Yi-Ling Lin, (yll@ibms.sinica.edu.tw).

### Materials availability

The plasmids generated in this study can be requested from the lead contact, Yi-Ling Lin, (yll@ibms.sinica.edu.tw).

### Data and code availability

• Plaque/focus images have been deposited at Mendeley and are publicly available as of the date of publication.
• This paper does not include original code.
• Any additional information required to reanalyze the data reported in this paper is available from the lead contact upon request.

## Acknowledgments

This study was supported by 10.13039/501100001869Academia Sinica and the 10.13039/100020595National Science and Technology Council (MOST 111-2320-B-001-021-MY3) in Taiwan. The authors acknowledge and thank the 10.13039/501100016004Institute of Biomedical Sciences (IBMS) Animal Core Facility for providing the necessary equipment and support for animal experiments. We also thank Dr. Chwan-Chuen King from the National Taiwan University College of Public Health for her valuable advice.

## Author contributions

Conceptualization, H.-Y.K., D.-Y.C., and Y.-L.L.; methodology, H.-Y.K. and G.-Y.Y.; investigation, H.-Y.K., Y.-T.L., H.-P.Y., Y.-Y.L., M.-T.C., Y.S., S.-S.D., and P.-J.C.; resources, Y.-L.Le.; writing, original draft, H.-Y.K.; writing, review and editing, D.-Y.C. and Y.-L.L.; visualization, H.-Y.K.; supervision, D.-Y.C., and Y.-L.L.; project administration, Y.-L.L.; funding acquisition, Y.-L.L.

## Declaration of interests

The authors declare no competing interests.

## STAR★Methods

### Key resources table

**Table:** 

REAGENT or RESOURCE	SOURCE	IDENTIFIER
**Antibodies**		

mouse anti-DENV NS1	Yao-Hong Biotechnology	YH0023
mouse anti-DENV E	Yao-Hong Biotechnology	YH0026
HRP-conjugated AffiniPure goat anti-mouse IgG	Jackson ImmunoResearch	Cat# 115-035-003; RRID: AB_10015289

**Bacterial and virus strains**		

DV-1202	This paper	GenBank: MG_599602

**Chemicals, peptides, and recombinant proteins**		

Methylcellulose	Sigma-Aldrich	CAS:9004-67-5
Isoflurane	Panion & BF biotech inc.	N/A
Rompun	Bayer Animal Health	N/A
Ketalar	Pfizer	N/A

**Critical commercial assays**		

NEBuilder HiFi DNA Assembly Master Mix	NEB	E2621
Q5 Site-Directed Mutagenesis Kit	NEB	E0554S
VECTOR VIP Peroxidase (HRP) substrate kit	Vector Laboratories	SK-4600
SYBR Green Master Mix	Applied Biosystems	Cat#4344463
Riboprobe *In Vitro* Transcription Systems kit	Promega	P1460
RNeasy mini kit	Qiagen	74104
KOD Hot Start DNA Polymerase kit	Merck/Novagen	71086
Streptavidin-Sepharose HP	Amersham Pharmacia	Cat#17-5113-01

**Deposited data**		

Plaque and Foci figures	This paper	http://www.doi.org/10.17632/ywb36s3cyc.2

**Experimental models: Cell lines**		

Human: 293T/17 cells	ATCC	CRL-11268
Monkey: Vero cells	ATCC	CRL-1587
Hamster: BHK-21	ATCC	CCL-10
Mosquito: C6/36 cells	ATCC	CRL-1660

**Experimental models: Organisms/strains**		

Mouse model (interferon-α/β and -γ receptor-knockout mice)	From National Health Research Institutes, Taiwan	4- to 6-week-old mice, AGB6
Mosquito model	From National Health Research Institutes, Taiwan	Female, *Aedes aegypti,* Higgs strain

**Oligonucleotides**		

Random hexamers	Promega	Cat# C1181
mFU1 sequence: 5'-TACAACATGATGGGAAAGCGAGAGAAAAA-3'	Chien et al.^62^	N/A
CFD2 sequence: 5'-GTGTCCCAGCCGGCGGTGTCATCAGC-3'	Chien et al.^62^	N/A
SNV-1073TC-F sequence: 5'-ACGACGATGGCGAAAAATAAAC-3'	This paper	N/A
SNV-1073TC-R sequence: 5'-Biotin-TGTTGGTCAGCTTTGCCTCTA-3'	This paper	N/A
SNV-8381CT-F sequence: 5'-CGCAACATCGGAATTGAAA-3'	This paper	N/A
SNV-8381CT-R sequence: 5'-Biotin-CTTGGTCATAGTGCCATGATGT-3'	This paper	N/A
SNV-8639AT-F sequence: 5'-GGACAACAGCGCGTTTTCA-3'	This paper	N/A
SNV-8639AT-R sequence: 5'-Biotin-CCATTCTGCCGTGATTTTCA-3'	This paper	N/A
SNP-1073TC-S sequence: 5'-ACCAACATTGGATTTTGAAC-3'	This paper	N/A
SNP-8381CT-S sequence: 5'-CATCGGAATTGAAAGTG-3'	This paper	N/A
SNP-8639AT-S sequence: 5'-ACAGCGCGTTTTCAA-3'	This paper	N/A

**Recombinant DNA**		

pGEM-T Easy Vector	Promega	A1360
pCMV-HDVr-SV40pA	From Dr. Eng Eong Ooi Lab, Duke-NUS Medical School	N/A

**Software and algorithms**		

Image J	Schneider et al.^63^	https://imagej.net
GraphPad Prism 9.0	La Jolla, CA, USA	https://www.graphpad.com

**Other**		

QuantStudio 3 Real-Time PCR System	Applied Biosystems	https://www.thermofisher.com/tw/zt/home/life-science/pcr/real-time-pcr/real-time-pcr-instruments/quantstudio-systems/
PyroMark Q24 system	Qiagen	https://www.qiagen.com/us/products/discovery-and-translational-research/pyrosequencing/pyromark-q24-and-advanced

### Experimental model and study participant details

#### Cell culture models

293T/17 cells (Human embryonic kidney cells, ATCC: CRL-11268) were cultured in Dulbecco’s Modified Eagle’s medium (DMEM, Gibco) supplemented with 10% fetal bovine serum (FBS, HyClone). Vero (African green monkey kidney cells, ATCC: CRL-1587) and BHK-21 (Baby hamster kidney fibroblast cells, ATCC: CCL-10) cells were grown in minimum essential medium (MEM, Gibco) with 10% FBS. All mammalian cells were maintained at 37°C with 5% CO_2_. On the other hand, C6/36 cells (midgut cells of *Aedes albopictus*, ATCC: CRL-1660) were grown in DMEM/MM medium (Mitsuhashi and Maramorosch insect medium, HiMedia) at 28°C without CO_2_. The culture medium was also supplemented with 1% L-glutamine (Gibco), non-essential amino acids (NEAA, Gibco), 1 mM sodium pyruvate, and a mixture of 1% penicillin/streptomycin (P/S, Gibco). The Mycoplasma-negative status of all cell lines was confirmed through routine testing by qPCR.

#### Mouse models

We carried out all animal experiments in compliance with guidelines for the care and use of laboratory animals as set forth by the Council of Agriculture, Executive Yuan, Taiwan. This protocol was approved by the Academia Sinica Institutional Animal Care and Use Committee (Protocol no. 21-12-1763). In order to minimize animal suffering, we used isoflurane anesthesia in mouse experiments. To evaluate the virulence of DENV-2 variants, we used 4- to 6-week-old male AGB6 mice (interferon-α/β and -γ receptor-knockout mice, National Health Research Institutes, Taiwan), and inoculated each mouse with 500 PFU through footpad injection. We collected blood samples from the submandibular area at 3 and 4 d.p.i., as well as serum samples for determining virus titers using FFA.

#### Mosquito infection and mouse-mosquito cross-species transmission model

We used female *Aedes aegypti* (Higgs) mosquitoes aged 7-14 days for this study. These mosquitoes were injected with 500 PFU of DENV via thoracic injection using the Nanojet II (Drummond), and then cultured at 28°C and 70% humidity with a 12-hour light/dark cycle and a 10% sucrose solution for 7 days. Infected mosquitoes were collected at 7, 14, and 21 days post-injection. To study virus transmission from mice to mosquitoes, we infected AGB6 male mice with 500 PFU of the Ia virus by tail vent injection and anesthetized them with Rompun (16 mg/kg, Bayer Animal Health) and Ketalar (100 mg/kg, Pfizer) on day 4 post-infection. Naïve female mosquitoes were allowed to take a blood meal from the mice, which were placed on a polyester mesh in a mosquito-housing cage. We collected blood samples from the mice after blood feeding. After feeding on the infected mice, we collected and incubated the engorged mosquitoes for 21 days for further analysis. We then quantified viral loads with RT-PCR, and detected minor variants with pyrosequencing.

### Method details

#### Construction and recovery of infectious clone-derived DENV-2 strains

We constructed the DENV-2 infectious clones using the Gibson assembly method modified from previous reports.^64^^,^^65^ In brief, we created the DNA library by inserting six synthesized DNA fragments based on clade II DENV-2 sequences (DV-1202, GenBank: MG_599602^10^) into pGEM-T Easy Vector (Promega). All infectious clones used in this study were based on a pCMV-HDVr-SV40pA vector, which contains a human cytomegalovirus (CMV) promotor, a hepatitis delta virus antigenomic ribozyme (HDVr), and a simian virus 40 polyadenylation signal (SV40-pA), kindly provided by Prof. Eng Eong Ooi (Duke-NUS Medical School, Singapore).^65^ We assembled the DNA fragments (including the six DENV-2 DNA fragments and one vector fragment) using NEBuilder HiFi DNA Assembly Master Mix (NEB). We then transfected the constructed plasmids into 293T/17 cells to recover the infectious clone-derived viruses. The viruses were amplified in C6/36 cells and checked for sequences with Sanger sequencing prior to use.^10^

We performed site-direct mutagenesis using the Q5 Site-Directed Mutagenesis Kit (New England Biolabs, NEB) to obtain required sequences. These clones were constructed to bear the designated amino acid changes, while maintaining the backbone of the clade II virus. These viruses, namely II-EEE (Ia), II-ELL (Ib), and II-LLL (II), were designated to represent each clade with their respective clade signatures. Additionally, we generated amino acid variations at three specific positions (E_46_, NS5_271_, and NS5_357_) in the infectious clone-derived DENV-2 viruses to explore the impact of each amino acid variant on the virus.

#### Viral growth kinetics in cell culture

We assessed viral growth kinetics by infecting a confluent monolayer of C6/36 and Vero cells with the infectious clone-derived DENV-2 viruses. The infection was conducted at a multiplicity of infection (MOI) of 0.1. Following infection, the cells were washed with phosphate buffered saline (PBS) and cultured in medium containing 2% FBS. Culture supernatant samples were collected at indicated time points (days post infection, d.p.i.) and quantitated using focus-forming assays (FFA).

#### Quantification of dengue virus

To determine the viral titers of the virus stocks, we conducted a plaque assay as previously described.^66^ We inoculated serially diluted samples in a 12-well plate containing a density of 2 × 10^5^ BHK-21 cells. After a 2h incubation, the cells were cultured with MEM containing 1% methylcellulose (MC, Sigma-Aldrich) and 2% FBS for 6 days at 37°C. Cells were then fixed with 10% formaldehyde in PBS and stained with 1% crystal violet solution. We counted and reported plaques as plaque forming units per ml (PFU/ml). We measured the plaque size for each strain as a mean plaque area (mm^2^) using the Image J software.^63^

We performed focus-forming assays (FFA) based on a protocol modified from a previous study.^59^ We infected Vero cells in 96-well plate (2 × 10^4^ cells per well) with serially diluted samples for 2 h, then overlaid the cells with medium containing 2% FBS and 1% MC. After 4 days of incubation, the cells were fixed with 10% formaldehyde in PBS and permeated with 1% Triton X-100. The infected cells were detected using mouse anti-DENV NS1 and E antibodies (YH0023 and YH0026, Yao-Hong Biotechnology) plus the secondary antibody, HRP-conjugated AffiniPure goat anti-mouse IgG (Jackson ImmunoResearch). We used VECTOR VIP Peroxidase (HRP) substrate kits (Vector Laboratories) to develop a violet signal after reacting with HRP. We then counted and reported viral titers as focus-forming units per ml (FFU/ml). We determined the focus size for each strain by measuring the mean foci area (mm^2^) using the Image J software.^63^

#### Measuring DENV RNA by RT-qPCR

The method for quantifying dengue RNA was modified from a previous report.^67^ We extracted virus RNA and synthesized cDNA with random hexamers (Promega). We quantified the levels of viral RNA using a qPCR assay that targeted the DENV RdRP gene with primers mFU1 (5′-TACAACATGATGGGAAAGCGAGAGAAAAA-3′) and CFD2 (5′-GTGTCCCAGCCGGCGGTGTCATCAGC-3′).^62^ The amplification produced a 266-nt product that was detected using the SYBR Green Master Mix and the QuantStudio 3 Real-Time PCR System (Applied Biosystems).^62^ The PCR product was cloned into a pGEMT vector, and the transcribed RNA was obtained by using the Riboprobe *In Vitro* Transcription Systems kit (Promega). This RNA product was serially diluted to produce a range of known concentrations and used as a quantification standard. The detection limit for the qPCR method was 10^2^ RNA copies/ml.

#### Replication capacity of DENV-2 epidemic strains

We determined the replication capacity by co-infecting cells with infectious clone-derived Ia (II-EEE) and II (II-LLL) viruses. We added a 1:1 mixture of Ia and II viruses at a MOI of 0.5 with 2% FBS medium to Vero and C6/36 cells seeded in 6-well plates for 2 h. Cells were first washed with PBS and maintained in 2% FBS medium for 5 days. We then determined the proportions of indicated SNVs through pyrosequencing. We performed all infection experiments in triplicate. We calculated the replicative index that measured the relative change of each SNV from its initial value based on a previous study.^15^ To compare between Ia and II at 5 d.p.i., we calculated the index as: ln[(proportion of late-epidemic SNVs at 5 d.p.i./ proportion of early-epidemic SNVs at 5 d.p.i.)/(proportion of late-epidemic SNVs at 0 d.p.i./ proportion of early-epidemic SNVs at 0 d.p.i.)]. This approach accounted for small errors in inputs by dividing the L/E ratio obtained on a given day (L/E SNV ratio on day 5) by the II/Ia ratio input (L/E SNV ratio on day 0). A replicative index greater than 0 indicates that the late-epidemic virus replicates more efficiently than the early-epidemic virus, whereas a value less than 0 signifies the opposite scenario.

#### Pyrosequencing of virus SNVs

Pyrosequencing targeting the three variants of the late epidemic signatures (SNV-1073T [E_46T_], SNV-8381C [NS5_271T_] and SNV-8639A [NS5_357E_]) was designed to monitor the dynamic changes of these SNVs. In brief, we extracted viral RNA from cell supernatant, mouse serum, or mosquito homogenate samples using the RNeasy mini kit (Qiagen). cDNA was synthesized using random hexamers (Promega) and amplified with biotin-labeled primers targeting distinct SNVs using KOD Hot Start DNA Polymerase kits (Merck/Novagen). The six primers targeting these three SNVs were SNV-1073TC-F: 5′-ACGACGATGGCGAAAAATAAAC-3′; SNV-1073TC-R: 5′-Biotin-TGTTGGTCAGCTTTGCCTCTA-3′; SNV-8381CT-F: 5′-CGCAACATCGGAATTGAAA-3′; SNV-8381CT-R: 5′-Biotin-CTTGGTCATAGTGCCATGATGT-3′; SNV-8639AT-F: 5′-GGACAACAGCGCGTTTTCA-3′, and SNV-8639AT-R: 5′-Biotin-CCATTCTGCCGTGATTTTCA-3′. The biotin-labeled PCR product was purified by Streptavidin-Sepharose HP (Amersham Pharmacia) and made into single stranded using a Pyrosequencing Vacuum Prep Tool. Three sequencing primers (SNP-1073TC-S: 5′-ACCAACATTGGATTTTGAAC-3′; SNP-8381CT-S: 5′-CATCGGAATTGAAAGTG-3′, and SNP-8639AT-S: 5′-ACAGCGCGTTTTCAA-3′) were used for pyrosequencing, which was performed by Mission Biotech (Taiwan) with the PyroMark Q24 system (Qiagen).

### Quantification and statistical analysis

The results are presented as means ± standard deviations (SD) of the three independent replicates if the sample size is not indicated. We analyzed the data using Student’s t-tests or one-way ANOVA. A p-value <0.05 was considered statistically significant.
